# Vulvovaginitis due to *Enterobius vermicularis* in a girl and epidemic enterobiasis in her family

**DOI:** 10.1002/ccr3.8902

**Published:** 2024-05-08

**Authors:** Lotfollah Davoodi, Eissa Soleymani, Ziaeddin Oladi, Shadi Shayesteh Azar, Fatemeh Parandin, Azadeh Mizani, Seyed Reza Mirbadie, Fatemeh Hajizadeh, Mahdi Fakhar

**Affiliations:** ^1^ Department of Infectious Diseases, Faculty of Medicine Antimicrobial Resistance Research Center, Mazandaran University of Medical Sciences Sari Iran; ^2^ Department of Parasitology, Toxoplasmosis Research Center Communicable Diseases Institute, Mazandaran University of Medical Sciences Sari Iran; ^3^ Department of Internal Medicine, Faculty of Medicine Mazandaran University of Medical Sciences Sari Iran; ^4^ Research Center for Environmental Determinants of Health (RCEDH) Health Institute, Kermanshah University of Medical Sciences Kermanshah Iran; ^5^ Department of Parasitology Pasteur Institute of Iran Tehran Iran; ^6^ School of Medicine Shahroud University of Medical Sciences Shahroud Iran; ^7^ Iranian National Registry Centre for Lophomoniasis and Toxoplasmosis Imam Khomeini Hospital, Mazandaran University of Medical Sciences Sari Iran

**Keywords:** enterobiasis, *Enterobius vermicularis*, mebendazole, vulvovaginitis

## Abstract

**Key Clinical Message:**

Here we present a case of a 4‐year‐old girl who suffered from vulvovaginitis caused by *Enterobius vermicularis*. All members of her family were also infected by this helminth. Treatment with mebendazole was administered to all family members and it was found that the entire family had been cured.

**Abstract:**

Vulvovaginitis, an inflammation of the vulvovaginal mucous membranes, is a common reason for pediatric gynecology consultations. One of the causes of this condition is a parasitic worm known as *Enterobius vermicularis* (*E. vermicularis*). In girls, adult worms can infiltrate the vagina and release eggs, leading to the development of vulvovaginitis. Furthermore, these worms have the ability to invade the endometrial cavity too. Here we present a case of a 4‐year‐old girl who suffered from vulvovaginitis caused by *E. vermicularis*. All members of her family were also infected by this parasitic helminth. In the vaginal sample, apart from the eggs, the female adult worm was observed under the microscope. Treatment with mebendazole was administered to all family members, and their progress was followed for a period of 3 weeks, during which it was found that the entire family had been cured. This patient experienced significant improvement in symptoms related to severe anxiety, nervousness, vaginal inflammation, itching, and vulvovaginitis caused by *E. vermicularis*. To prevent infection by *E. vermicularis*, it is crucial to disinfect underwear and bed sheets. In kindergartens, the spread of this parasite should not be underestimated, and asymptomatic individuals who have been exposed to infected persons should receive treatment to prevent an epidemic. Maintaining cleanliness and hygiene, especially after using the toilet, is of the most importance, particularly for girls who are more susceptible to *E. vermicularis* infection. Additionally, it is essential for all family members to be aware of the transmission routes of this parasite.

## 1 INTRODUCTION

Vulvovaginitis is the inflammation of the vulvovaginal mucous membranes^1^ and is a common reason for pediatric gynecology consultations.^2^ Prepubertal girls often experience gynecologic issues characterized by symptoms such as vulvovaginal itching, discharge, irritation, burning, or skin changes. The development of these symptoms is influenced by anatomical, physiological, and behavioral factors specific to this age group.^3^ *Streptococcus pyogenes*, *Haemophilus influenzae* and *Enterobius vermicularis* (*E. vermicularis*) are commonly identified as the predominant pathogens, while fungal and viral infections have lower occurrence rates.^4^ Symptoms such as genital discomfort or a burning sensation during urination are frequently observed in cases of vulvovaginitis. This condition, more prevalent in prepubescent girls, can be caused by a deficiency of estrogen and poor local hygiene, which can lead to infection of the vaginal mucosa. While precise data on its prevalence is lacking, these predisposing factors are known to contribute to the development of vulvovaginitis.^3^

In addition, favor factors of development of this disease include local alkaline pH, thin labia minora and reauctioned estrogen stimulus during the prepubertal period results in thinning of the vulvovaginal epithelium. Among prepubertal girls, the most common clinical presentation is nonspecific vulvovaginitis caused by endogenous vaginal flora.^5^ One of the most agents of vulvovaginitis is a parasite called *E. vermicularis*.^4^ This worm exhibits the most extensive geographical distribution among helminths.^6^ Its induced infection is a global phenomenon and is recognized as the most prevalent form of helminth infection.^7^ This condition is prevalent across all age groups and socioeconomic backgrounds, although it is particularly widespread among children between the ages of five and fourteen.^8^, ^9^ It is important to note that parasitic infections even in children may lead to malnutrition and decreased learning abilities.^10^ Embryonated eggs can be detected on various surfaces such as fingernails, clothing, house dust, and other objects. Once these eggs are ingested, they undergo hatching within the stomach, giving rise to larvae.

These larvae then make their way to the cecum, where they undergo further development and eventually reach adulthood as pinworms, measuring approximately 1 cm in length. The gravid adult female worms exhibit a nocturnal migration to the perianal region, where they lay a substantial number of eggs, up to 11,000 in total. These eggs become infective within a relatively short period of time, approximately 6 h after being deposited. The lifespan of *E. vermicularis* typically ranges from 11 to 35 days.^8^ Transmission of the infection takes place via direct transmission from an infected individual through the oral‐anal route, or through the dispersal of airborne eggs from contaminated clothing or bed linen. Upon ingestion, the eggs hatch and release larvae within the intestine.^11^

Adult worms in girls may also infiltrate the vagina to release eggs and consequently leading to the development of vulvovaginitis. In 1980, Vaughan reported one of the first enterobiasis in direct observation of vaginal region.^9^, ^12^ Moreover, these worms possess the ability to invade the endometrial cavity, thereby inducing endometritis and salpingitis in affected patients.^9^ For diagnosis collection of eggs from infected area (anus or vagina) can be achieved through the use of the cellophane swab or scotch tape swab method. stool examination not be a reliable means of detecting eggs.^13^ Cases have been documented where this worm has traversed the entire reproductive system and penetrated the peritoneal cavity via the fallopian tubes.^9^ In this paper, we present the case of a 4‐year‐old girl who suffered from vulvar itching and vulvovaginitis caused by *E. vermicularis*. Her family was also infected by this parasitic helminth.

## 2 CASE HISTORY/EXAMINATION

An anxious and very nervous 4‐year‐old girl that suffered from severe vulvar itching and burning was referred to Razi Hospital in Qaemshahr town, northern Iran. According to her parents, her older sister, who was 6 years old, refrained from playing with him due to the fear of exposure of infection. The patient's mother and sister did not have any sing and symptoms of enterobiasis, and only her father had a slight itching of the anus.

## 3 METHODS (DIFFERENTIAL DIAGNOSIS, INVESTIGATIONS AND TREATMENT)

**FIGURE 1:**
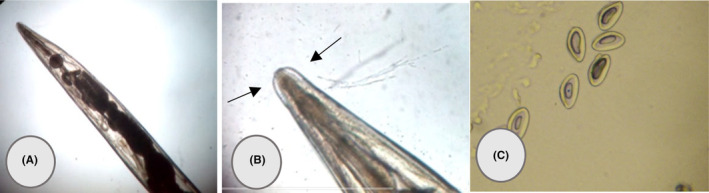
Vulvovaginitis by *Enterbios vermicularis* in the vagin of a 4‐year‐old girl. (A) Adult female associated with full of eggs uterus. (B) Cervical alae (arrows). (C) The eggs.

We doubted *E. vermicularis* and gave her scotch tape for sampling. We asked her parents to take samples from both her vagina and anus. To our surprise, in addition to the eggs, we observed the female adult worm under the microscope (Figure 1). We asked the mother, father, and older sister to take samples using scotch tape the following morning after waking up and before using the toilet. Numerous eggs were found in the samples from all three individuals, which were identified as belonging to *E. vermicularis*. Interestingly, the mother and older sister had *E. vermicularis* infections without displaying any symptoms. Treatment with mebendazole (100 mg as a single dose, repeated after 2 weeks) was administered to all family members. After monitoring the cases for 3 weeks, it was observed that the entire family was cured.

In the case of the 4‐year‐old girl, her symptoms such as severe anxiety, nervousness, vulvar itching, vaginal inflammation, and vulvovaginitis caused by *E. vermicularis* were completely resolved.

## 4 DISCUSSION

Infection caused by *E. vermicularis* outside of the intestines is infrequent, with the female genital tract being the primary site of involvement. Ectopic enterobiasis has been reported in various regions of the female genital tract, including the uterus, ovary, vagina, fallopian tubes, and pelvic peritoneum.^14^ Cytological examinations have occasionally identified intestinal parasites that are responsible for vaginal enterobiasis. While enterobiasis is commonly believed to be asymptomatic or to only cause minor symptoms such as perianal itching, it is important to note that this parasite has the potential to cause severe and potentially life‐threatening illnesses, and in some cases, even death.^6^ Non‐gastrointestinal manifestations of *E. vermicularis* are such as pruritus vulvae, urinary tract infections, postmenopausal bleeding, epididymitis, pelvic mass, tubo‐ovarian abscess, and generalized peritonitis.^6^, ^11^ The invasion of the endometrial cavity by *E. vermicularis* can result in the development of endometritis and salpingitis.^9^ It is crucial to avoid mistaking them for other types of parasitic ova, pollen grains, or tainted plant cells.^9^, ^15^ The dimensions of *E. vermicularis* eggs are 55 μm in length and 25 μm in width, with the width being half of the length.^9^ The presence of these characteristics aids in differentiating from other possible impurities that may be present in vaginal specimens, such as fibers, plant matter, fungi, and so on.^16^ Studies has shown that *E. vermicularis* has the ability to invade the urinary and vaginal tracts in female children, resulting in the development of vulvovaginitis.^17^ Our case was a 4‐year‐old girl who had severe vaginal itching caused by *E. vermicularis* and was highly anxious and nervous. The entire family was also infected with this parasite. enterobiasis is a widely recognized form of parasitic infection that affects children^18^ with a prevalence about 17.2% in Iran.^19^, ^20^

## 5 CONCLUSION

Enterobiasis, is a significant concern due to its easy spread within families and children's groups.^21^ The primary mode of transmission is through the fecal‐oral route, with eggs remaining viable on clothing and bedding for up to 3 weeks.^8^, ^22^ To combat this, disinfecting underwear and bed sheets is crucial, especially in kindergartens where the parasite can easily spread. Asymptomatic individuals exposed to infected persons should be treated, and regular scotch tests in kindergartens can help detect and prevent further transmission. Treating all family members simultaneously is essential, along with educating them on preventive measures.^23^ Maintaining cleanliness, particularly after toileting, is vital to prevent infection, especially in girls who are more susceptible to complications like vulvovaginitis. Awareness of transmission routes and proactive measures are key in controlling and preventing the spread of enterobiasis.^21^

## AUTHOR CONTRIBUTIONS

**Lotfollah Davoodi:** Project administration. **Eissa Soleymani:** Investigation; methodology; writing – review and editing. **Ziaeddin Oladi:** Writing – review and editing. **Shadi Shayesteh Azar:** Investigation. **Fatemeh Parandin:** Investigation. **Azadeh Mizani:** Investigation. **Seyed Reza Mirbadie:** Investigation. **Fatemeh Hajizadeh:** Investigation. **Mahdi Fakhar:** Project administration; supervision; writing – review and editing.

## FUNDING INFORMATION

None.

## CONFLICT OF INTEREST STATEMENT

The authors declare that they have no conflict of interest.

## CONSENT

Written informed consent was obtained from the patient to publish this report in accordance with the journal's patient consent policy.

## Data Availability

The data is accessible through the corresponding author and can be obtained upon request.
